# Langerhans cell histiocytosis: A case report with oral manifestations and the role of pediatric dentists in the diagnosis

**DOI:** 10.1002/ccr3.2726

**Published:** 2020-02-15

**Authors:** Eman Hussein Hammouri, Hala Antoun Sweidan, Omar AShokaibi, Leen Al Omari

**Affiliations:** ^1^ Jordanian Royal Medical Services Amman Jordan

**Keywords:** dentists, floating teeth, histiocytosis X, Langerhans cell histiocytosis

## Abstract

Langerhans cell histiocytosis (LCH) is a benign disease that behaves malignantly. Early recognition and treatment of oral manifestation of LCH by pediatric dentist and other medical specialties is important to prevent further organ damage.

## Graphical abstract

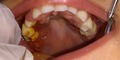

## 1 INTRODUCTION

Langerhans cell histiocytosis (LCH) is a rare hematological disease that usually affects children.^1^ Although the underlying pathological cause is still to be identified, the disease is characterized by abnormal proliferation of bone marrow‐derived histiocytes known as Langerhans cells.^2^, ^3^, ^4^ The combination of the rarity of the disease (it affects fewer than five people per million), the diversity of its clinical manifestations, and the need for histopathological diagnosis lead to difficulty in diagnosis.^1^, ^2^, ^3^, ^5^, ^6^, ^7^ The histiocytes may infiltrate and damage single or multiple organs particularly bones.^8^, ^9^, ^10^, ^11^, ^12^ LCH occurs most commonly in the head and neck region, and when jaws and gingiva are affected, the patients may have nonspecific symptoms and signs such as gingival necrosis and hypermobility of the teeth (floating teeth) that may lead to misdiagnosis.^4^, ^7^, ^13^, ^14^, ^15^, ^16^, ^17^ This article discusses the oral manifestations of LCH, radiological findings, histological features, review of literature, and the role of dentists in the diagnosis.

## 2 CASE REPORT

**Figure 1:**
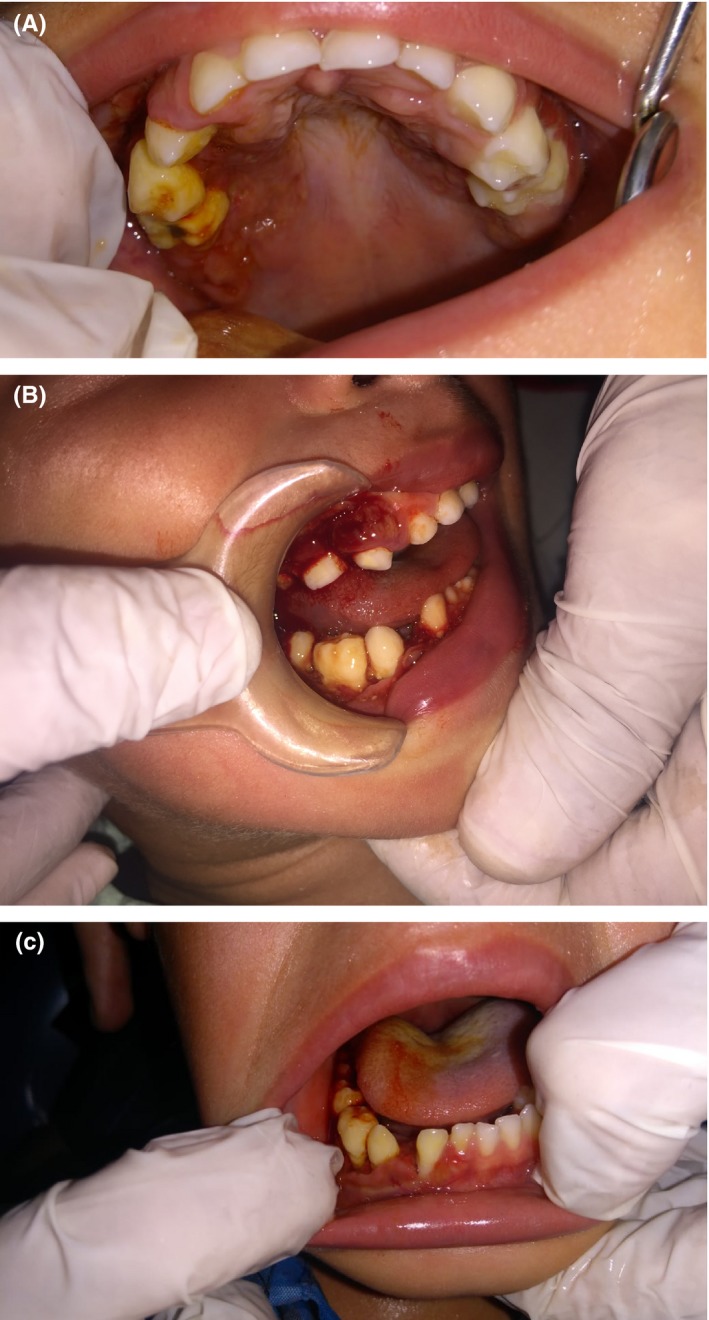
A, Palatal necrosis adjacent to the first and second upper right primary molars covered with plaque and calculus. B, Gingival inflammation facing the lower right first, second primary molars, and canine along with food debris. C, Gingival recession and inflammation related to the lower right canine with increased space and tilting of the canine

**Figure 2:**
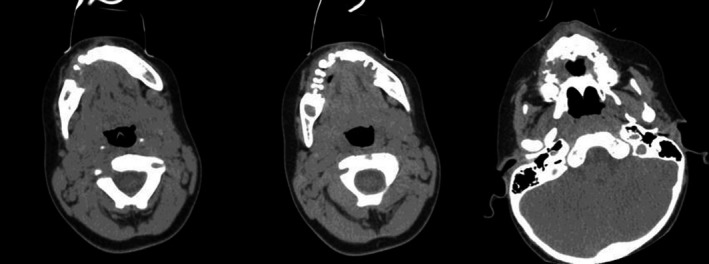
Head and neck CT scan shows irregular destructive radiolucent lesions in the mandible and the maxillary bones with soft tissue component

**Figure 3:**
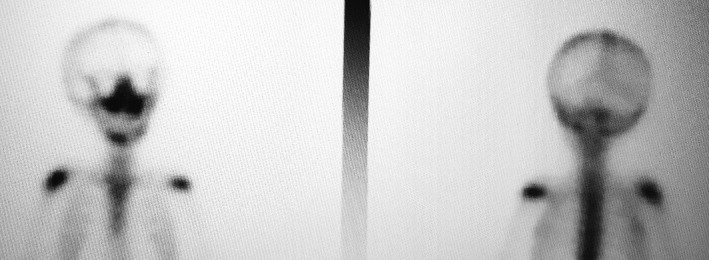
Tc99m‐MDP Bone Isotope scan (WB) after treatment showed increased radiotracer uptake within the mandible, and the remainder of the skeleton was within normal limit

**Figure 4:**
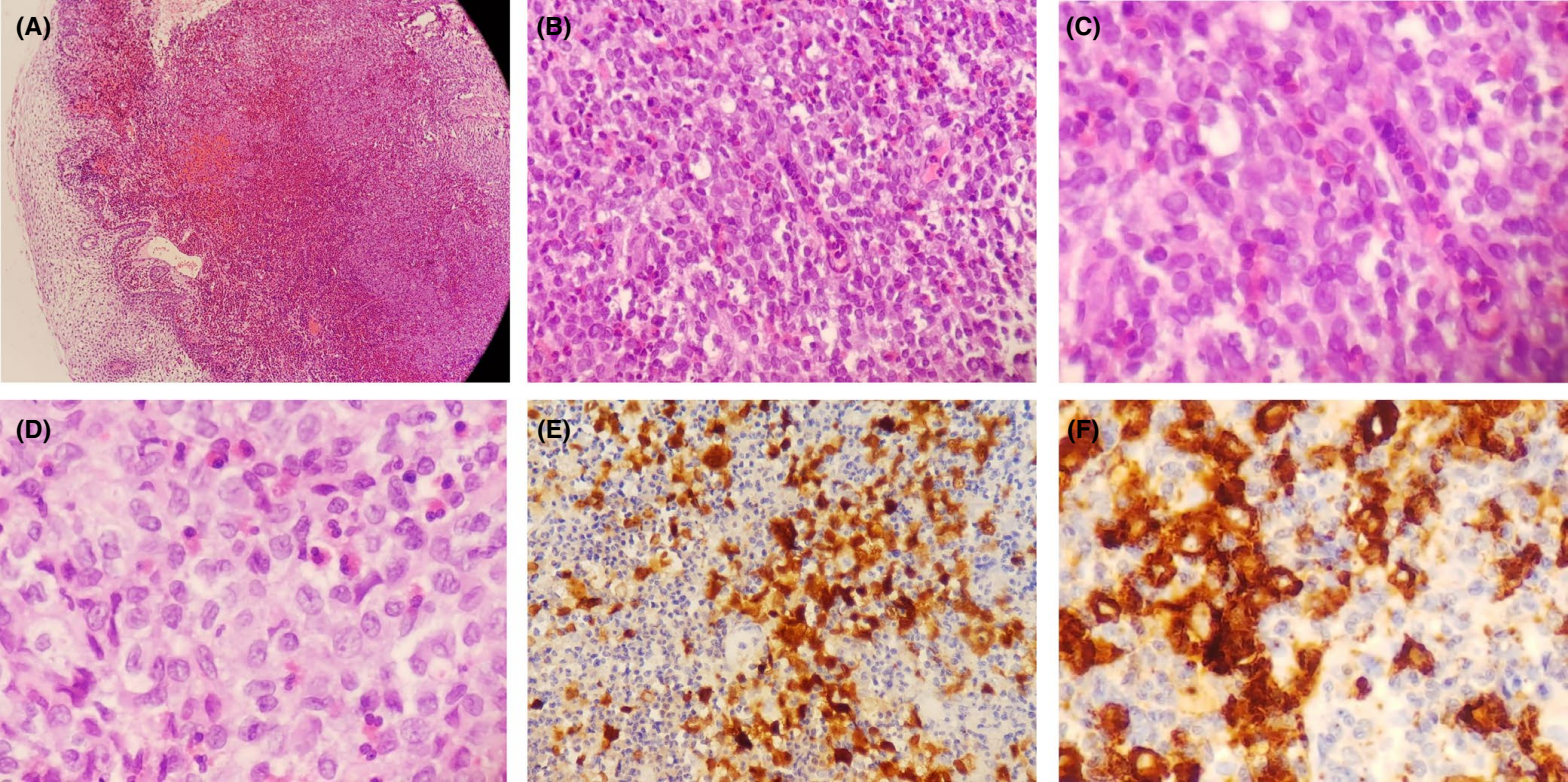
A, Histopathological gingival infiltration by sheets of large histiocytosis with coffee‐bean‐like nuclei and typical and atypical mitoses in a background rich in eosinophils, some lymphocytes foam cells, and plasma cells. B, Histopathological gingival infiltration by sheets of large histiocytosis with coffee‐bean‐like nuclei and typical and atypical mitoses in a background rich in eosinophils, some lymphocytes foam cells, and plasma cells. C, Histopathological gingival infiltration by sheets of large histiocytosis with coffee‐bean‐like nuclei and typical and atypical mitoses in a background rich in eosinophils, some lymphocytes foam cells, and plasma cells. D, Histopathological gingival infiltration by sheets of large histiocytosis with coffee‐bean‐like nuclei and typical and atypical mitoses in a background rich in eosinophils, some lymphocytes foam cells, and plasma cells. E, Immune reactivity of tumor cells for S‐ 100 and CD1a. F, Immune reactivity of tumor cells for S‐ 100 and CD1a

A 2‐year‐old‐male patient was referred to the pediatric dental clinic at Prince Rashid Bin Al Hassan military hospital in the north of Jordan for treatment of oral ulcerations, bleeding gingiva, and difficulty in eating. Oral examination revealed halitosis, gingival necrosis and ulcerations, oozing of blood from the gingiva, and floating teeth, Figure 1. Oral panoramic tomography was difficult to obtain due to a lack of cooperation at this age. Full blood count, erythrocyte sedimentation rate (ESR), thrombophilia screening, blood chemistry, and pediatric consultation were requested to exclude leukocyte adhesion deficiency (LAD), hypophosphatasia, scurvy, and leukemia. Head and neck CT scan was performed under general anesthesia and showed irregular destructive radiolucent lesions in the maxillary and mandibular bones with soft tissue component, Figure 2. Tc^99m^‐MDP Bone Isotope scan showed increased radiotracer uptake within the mandible and maxilla, while the remainder of the skeleton was within normal limits. Figure 3 showed Tc^99m^‐MDP Bone Isotope scan (WB) after treatment. An incisional biopsy was taken from a lesion in the superficial alveolar bone, and surgical curettage was done for other bone lesions. Histopathological examination revealed gingival infiltration by sheets of large histiocytosis having coffee‐bean‐like nuclei and typical and atypical mitoses accompanied by numerous eosinophils, some lymphocytes foam cells, and plasma cells. The histiocytes are large and polygonal with an eosinophilic cytoplasm. The tumor cells are immune‐reactive for S‐100 and CD1a, Figure 4. The definitive diagnosis was Langerhans cell histiocytosis, and the patient was referred to the pediatric oncology department for further investigation and management.

## 3 DISCUSSION

Langerhans cell histiocytosis, previously known as histiocytosis X, is a rare and destructive disease that may clinically and radiologically mimic malignant disorders.^1^, ^7^, ^12^, ^14^ A high index of suspicion is needed to consider the diagnosis of LCH particularly in pediatric patients with unclear osteolytic lesions.

Although the disease is usually encountered in patients between 1 and 10 years of age with male predominance, recently reported cases described LCH in a five‐month‐old infant^17^ and patients aged between 17 and 63 years old. The present article discusses the case of a 2‐year‐old male.^2^, ^7^, ^9^, ^12^, ^14^, ^15^, ^18^

The disease may be present with solitary or multiple osteolytic lesions in single or multiple bones with the head and neck being the most commonly affected, particularly the jaws.^17^ Oral manifestations may include gingivitis, periodontitis, tooth rotation or loss, and malocclusion. In the present case, the osteolytic lesions were encountered in the maxillary and mandibular bones causing hypermobility of the teeth (floating teeth).^15^, ^16^, ^17^

Diabetes insipidus is a common LCH complication related to the central nervous system due to pituitary gland involvement and may need lifelong hormonal replacement.^18^

Recurrent mouth ulceration and gingival necrosis are nonspecific signs that can be caused by various disorders such as leukocyte adhesion deficiency (LAD), hypophosphatasia, scurvy, leukemia, and medication‐related osteonecrosis of the jaw, but when there is hypermobility of teeth, then bone destruction should be suspected and radiological investigation should be requested and this emphasizes the rule of a pediatric dentist in diagnosis of systemic disorders such as LCH.^19^ The definitive diagnosis of LCH is made by histopathology where sheets of large histiocytosis with longitudinal groove (coffee‐bean‐like) nuclei and typical and atypical mitoses in a eosinophils rich background, and immunohistochemical stains are of utmost importance in highlighting reactivity to S‐100 and CD1a.^15^, ^20^, ^21^, ^22^ A monoclonal antibody against CD207 (langerin) is a highly specific and sensitive protein that is needed for formation of the Birbeck‐Broadbent granules.^23^ FDG‐PET scan is required to exclude intense radiotracer activity in other sites of the body and to assess the response for treatment; in the present case, Tc^99m^‐MDP Bone Isotope scan was used and showed increased radiotracer uptake within the maxillary and mandibular bones only.^24^ A previous molecular study showed a six base pair deletion in exon 3 of the *MAP2K1* gene (p.E102_I103del).^15^ A genetic study was not ordered in this case.

Because of the clinical variability of the disease and lack of standard diagnostic tools and evaluation, optimal treatment of LCH is still controversial and depends on the age of the patient and the extent of the disease. Small solitary osteolytic lesions or single system disease exhibiting benign course is usually treated with surgical curettage followed by a local injection of steroids for the remission of symptoms.^15^, ^25^, ^26^ However, radiotherapy or chemotherapy, or both are needed in case of multiple organ involvement, a big solitary lesion, or recurrence.^27^, ^28^ In the present case, surgical curettage was done at the time of biopsy and the patient was referred for chemotherapy treatment by the pediatric oncology department. The patient showed significant clinical and radiological improvement with chemotherapy (Vinblastine, Methotrexate, and oral prednisolone). Close follow‐up is mandatory to rule out the recurrence of the disease, which depends on the degree of tissue involvement and method of treatment. The ten‐year survival rate for a single bone involvement has been reported to be 100%.^7^, ^29^

## 4 CONCLUSIONS

The oral manifestations may be the key to the diagnosis of Langerhans cell histiocytosis. In order to identify and manage systemic illnesses such as LCH, dentists should bridge the gap with other medical specialists.

The following points highlight the importance of this case study to a pediatric dentist:

- A thorough history and oral examination by a pediatric dentist may aid in determining the underlying cause of oral manifestations and allow for earlier intervention by other medical subspecialties.
- Correctly timed diagnosis by a pediatric dentist saves time, expenses, and limits the extent of the disease.
- It reflects the role of a pediatric dentist in improving the quality of patients’ lives and easing their families’ sufferings by treating oral manifestations of serious systemic illnesses.

## CONFLICT OF INTEREST

None declared.

## AUTHORS’ CONTRIBUTIONS

EHH: was the corresponding author, had substantial contributions to the conception, design of the work, acquisition, analysis, interpretation, and drafted the work and revised it, and served as the pediatric dentist who diagnosed the case. HAS: had substantial contributions to the conception, design of the work, acquisition, analysis, interpretation, and revision, and served as the treating pediatrician. OA‐S: had substantial contributions to the conception, design of the work, and revision, and served as the histopathologist who read the slides and diagnosed the case. LAO: had substantial contributions to the conception, design of the work, acquisition, analysis, and interpretation, and served as the pediatric dentist. All authors: approved the submitted version and agreed to be personally accountable for the author's own contributions and to ensure that questions related to the accuracy or integrity of any part of the work, even ones in which the author was not personally involved, are appropriately investigated, resolves, and the resolution documented in the literature.
